# Retraction: ADAMTS13 maintains cerebrovascular integrity to ameliorate Alzheimer-like pathology

**DOI:** 10.1371/journal.pbio.3002036

**Published:** 2023-03-09

**Authors:** The PLOS Biology Editors

After this article [1] was published, concerns were raised, including some by the authors, about similarities between micrographs and western blots in Figs 2, 3, 5, 6, S4, and S5 in article [1] and micrographs and western blots in Figures 1, S2-S4 in a *Blood* article [2]. Specifically:

- The 12m *APPPS1* panel in Fig 2A of [1] appears duplicated in the Gal-3+DMSO, 40K Dextran panel in Fig S4B in [2], when rotated 180 degrees.
- The 12 month β-actin panel in Fig 2E in [1] appears duplicated in the 6 month β-actin panel in Fig S2B in [1].
- The 6m *APPPS1 Adamts13*^*-/-*^ panel in Fig 3B in [1] appears duplicated in the AAV-Control panel in Fig 6H in [1].
- The lower right quadrant of the 2000K Dextran, 6m WT panel in Fig 3B of [1] appears duplicated in the lower right quadrant of the WT Ischemia panel in Fig 1E of [2], when rotated 180 degrees.
- In Fig 5H in [1], the right side of the *APPPS1* panel appears duplicated in the left side of the *APPPS1 Adamts13*^-/-^ panel.
- The AAV-Control panel in Fig 6C in [1] appears duplicated in the rGal-3, 40K Dextran panel in S4B in [2], when reflected vertically.
- In Fig S4 in [1]:
  ○ The lower half of the 6m WT panel appears duplicated in the upper half of the 12 m WT panel.
  ○ The 6m *Adamts13*^*-/-*^ panel appears duplicated in the 12m *APPPS1* panel.
- The 6m *APPPSS1 Adamts13*^*-/-*^ panel in Fig S5A in [1] appears duplicated in the *Adamts13*^*-/-*^ panel in Fig S3A in [2], when rotated 90 degrees.

During editorial follow up on these issues, images stated to belong to the original experiments were provided by the authors for all figures of concern in [1]. The corresponding author stated that multiphoton and confocal microscope imaging experiments were running for the two articles ([1] and [2]) simultaneously, and that this led to unintentional errors and the inclusion of a number of incorrect images in [1] and [2]. Replacement panels for the stated incorrect images in [1] were provided, but given the extent of the duplications, the *PLOS Biology* Editors consider these insufficient to address the concerns. The corresponding author also stated that the following panels in [1] are incorrect:

- The 6m *Adamts13*^*-/-*^ panel in Fig 3B, as the image was taken from the same 6m *APPPS1* mouse as for the 6m *APPPS1* panel in Fig 3B;
- The 12m WT panel in Fig 3B, as the image was taken from the same ischemic mouse as for the WT Ischemia panels in Figs 1B and E of [2];
- The 2 month β-actin panel in Fig S2B, as this panel was reflected horizontally with two lanes incorrectly included;
- The 12 month β-actin panel in Fig S2B, as two lanes were incorrectly included and all four lanes mislabeled;
- The 2m *APPPS1* panel in Fig S4.

Whilst underlying data were provided, the extent of the data reporting issues and image concerns call into question the reliability of the conclusions and the overall integrity of data reporting. The *PLOS Biology* Editors therefore retract this article.

HL, XY, LW, ZW, HX, YZ, YW, MJS, XB, LK, BQZ, WF, SC, RW, YS, YM, and YC did not agree with retraction. JZ, HT, LL, and KZ either did not respond directly or could not be reached.

XY, LW, YC, ZW, HX, YZ, YW, MJS, XB, LK, BQZ, WF, SC, RW, YS, YM, and YC stand by the article’s findings.

YC apologizes for the issues with the published article.

Owing to the concerns about similarities with previously published content [2], published 2017 by The American Society of Hematology and which is not offered under a CC-BY license, the 12m *APPPS1* panel of Fig 2A, the 6m WT panel of Fig 3B, the AAV-Control panel of Fig 6C, and the 6m *APPPS1 Adamts13*^*-/-*^ panel in Fig S5A are excluded from this article’s [1] license. At the time of retraction, the article [1] was republished to note these exclusions in the Figs 2A, 3B, 6C, and S5A legends and the article’s copyright statement.
